# Depression, Mindfulness, and Psilocybin: Possible Complementary Effects of Mindfulness Meditation and Psilocybin in the Treatment of Depression. A Review

**DOI:** 10.3389/fpsyt.2020.00224

**Published:** 2020-03-31

**Authors:** Kristin Heuschkel, Kim P.C. Kuypers

**Affiliations:** Department of Neuropsychology and Psychopharmacology, Faculty of Psychology and Neuroscience, Maastricht University, Maastricht, Netherlands

**Keywords:** depression, mindfulness meditation, psilocybin, psychedelics, review

## Abstract

Depression is a major public health problem that affects approximately 4.4% of the global population. Since conventional pharmacotherapies and psychotherapies are only partially effective, as demonstrated by the number of patients failing to achieve remission, alternative treatments are needed. Mindfulness meditation (MM) and psilocybin represent two promising novel treatments that might even have complementary therapeutic effects when combined. Since the current literature is limited to theoretical and empirical underpinnings of either treatment alone, the present review aimed to identify possible complementary effects that may be relevant to the treatment of depression. To that end, the individual effects of MM and psilocybin, and their underlying working mechanisms, were compared on a non-exhaustive selection of six prominent psychological and biological processes that are well known to show impairments in patients suffering from major depression disorder, that is mood, executive functioning, social skills, neuroplasticity, core neural networks, and neuroendocrine and neuroimmunological levels. Based on predefined search strings used in two online databases (PubMed and Google Scholar) 1129 articles were identified. After screening title and abstract for relevance related to the question, 82 articles were retained and 11 were added after reference list search, resulting in 93 articles included in the review. Findings show that MM and psilocybin exert similar effects on mood, social skills, and neuroplasticity; different effects were found on executive functioning, neural core networks, and neuroendocrine and neuroimmune system markers. Potential mechanisms of MM’s effects are enhanced affective self-regulation through mental strategies, optimization of stress reactivity, and structural and functional adjustments of prefrontal and limbic areas; psilocybin’s effects might be established *via* attenuation of cognitive associations through deep personal insights, cognitive disinhibition, and global neural network disintegration. It is suggested that, when used in combination, MM and psilocybin could exert complementary effects by potentiating or prolonging mutual positive effects, for example, MM potentially facilitating psilocybin-induced peak experiences. Future placebo-controlled double-blind randomized trials focusing on psilocybin-assisted mindfulness-based therapy will provide knowledge about whether the proposed combination of therapies maximizes their efficacy in the treatment of depression or depressive symptomatology.

## Introduction

Depression or major depressive disorder (MDD) is a common mood disorder and major cause of disability worldwide. Approximately 4.4% of the global population is affected by this condition, with wide-ranging variations across gender, age, and nationality (1). Typical symptoms include depressed mood, anhedonia, fatigue, feelings of worthlessness or guilt, changes in appetite, weight, and sleep, psychomotor retardation or agitation, executive deficits, and suicidal ideation (2). These are thought to originate from a complex interplay of psychological and biological factors (3).

Psychological factors that underlie the pathology of MDD comprise deficiencies on an emotional, cognitive, and social level (3). Negative thinking patterns paired with inadequate emotion regulation and excessive rumination have been implicated in the maintenance of depressed mood (4, 5). The aforementioned combination of these three psychological processes further promotes cognitive rigidity, as evident from underperformance in executive functioning tests measuring for example task-switching, working memory (WM), attention, and inhibitory control (4, 6–8). To exemplify, depressed patients take more time to adapt to new rules in the Wisconsin Card Sorting Test and show attentional and memory deficits predominantly in the context of positive affective stimuli (9, 10).

The emotional and cognitive deficiencies accompanying MDD have an impact on interpersonal competencies as well (11, 12). Not only do depressed people show differences in dispositional empathy compared to controls, with for example higher personal distress (13, 14), they also demonstrate shortcomings in communication skills, which might, for example, be expressed in an inanimate body language and bias toward negative facial expressions and conversational contents (12, 15). Additionally, they tend to seek excessively for approval and negative feedback, which may verify their negative self-image (12). Such poor social skills along with self-centered introversion provoke conflicts within the social environment, which pose stressors that crucially contribute to the perpetuation of depressive symptoms (16).

Biological factors that pertain to the characteristics of MDD range from neural imbalances to signaling dysregulations, partly grounded in genetic predispositions (3). Neuroplasticity, a crucial neural mechanism that entails structural and functional brain adaptations in response to altered environmental circumstances, is impaired in individuals with depression, as indicated by abnormally low levels of brain-derived neurotrophic factor (BDNF), the latter being related to hippocampal and prefrontal atrophy in MDD (17, 18). Deficiencies in MDD BDNF levels might originate from epigenetic factors, such as stress exposure (19, 20). A meta-analysis showed that clinical changes in depression were related to BDNF levels, and suggested a role for neuroplasticity in the improvement of symptoms (21)

Another biological disruption in MDD concerns the imbalances between functionally connected fronto-limbic and thalamo-cortical networks, which could further contribute to the maintenance of negative and rigid thinking patterns (22). More precisely, MDD is associated with hyper-connectivity within the default mode network (DMN), a system of brain areas engaged during rumination (22, 23). The DMN works in close accordance with the central executive network (CEN), a group of brain regions involved in WM and goal-directed behavior (24, 25), and the salience network (SN), which mediates the activity of the DMN and CEN according to the saliency of external or internal stimuli (26). In MDD, both the SN and CEN are intrinsically hypo-connected. In addition, the SN is generally hyper-connected to the DMN, while being over-responsive to negative emotional stimuli. This state relates to emotional over-reactivity in depressed patients. The CEN, on the other hand, is under-reactive to negative affective stimuli and its connections to the DMN and SN are weakened compared to that of healthy controls. This disrupted biological brain pattern is linked to deficits in executive functioning (27, 28).

MDD also features dysregulations within the hypothalamic-pituitary-adrenal (HPA) axis, a circuit within the neuroendocrine system that plays a central role in the regulation of stress and immune responses. Hypersecretion of cortisol and impaired negative feedback result in chronically elevated cortisol levels, which increase the vulnerability to stressors, cause disruptions in monoamine and immune systems, and ultimately promote the emergence of depressive symptoms (3, 18, 29). The inadequate HPA responsivity in MDD is further marked by a diminished cortisol awakening response (CAR), as opposed to healthy people who demonstrate steeply elevated cortisol levels within the first 30 min upon awakening (30, 31). Moreover, abnormally high levels of pro-inflammatory cytokines, such as interleukin 6 (IL-6), can be found in depressed patients (32), which is why theories link depression to inflammation (33). IL-6 has a stimulating effect on the HPA axis, and mediates BDNF levels (34, 35).

**Figure 1:**
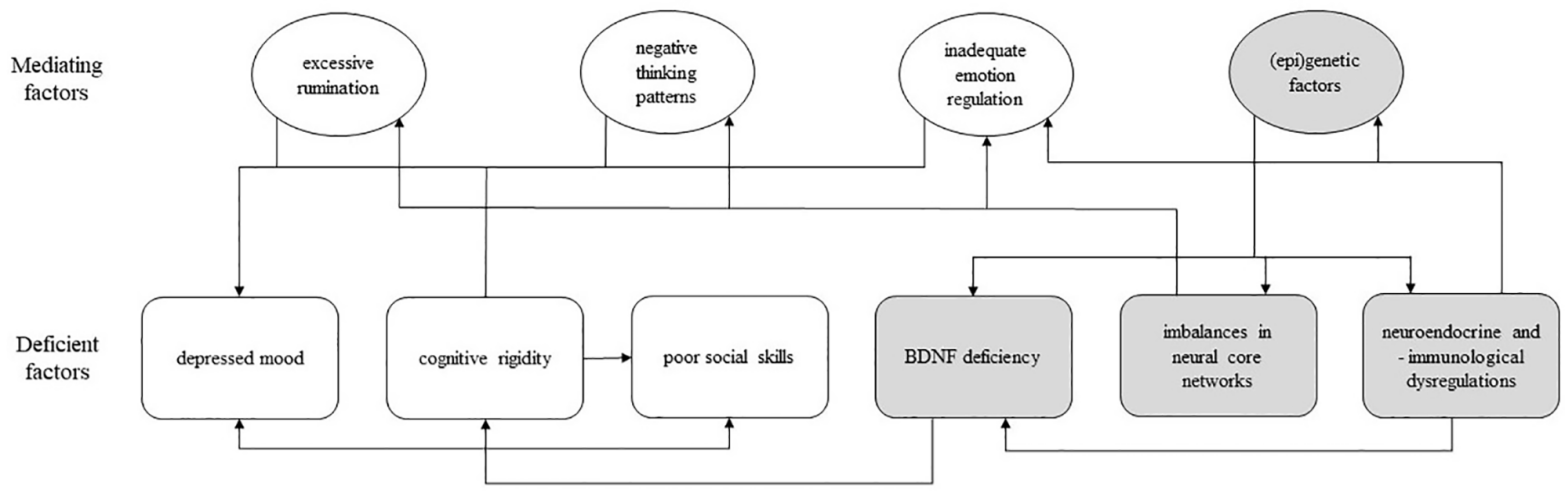
A model of psychological and biological deficiencies associated with major depressive disorder; rounded square-shaped box, deficient factor(s); oval-shaped box, mediating factor(s); white box, psychological factor; gray box, biological factor; arrow, unidirectional influence; BDNF, brain-derived neurotrophic factor.

The outlined socio-cognitive and biological deficiencies can be categorized into six non-exhaustive broad factors, i.e., mood, executive functioning, social skills, neuroplasticity, neural core networks, and neuroendocrine and neuroimmunological factors. Although these factors seem to play a causal role in the symptomology of MDD to differing degrees, the precise etiology of depression is not known and cannot be delineated from the current evidence. The six individual factors presented here appear to influence each other in a circular, perpetuating manner, as illustrated in **Figure 1**. Thus, the modulation of one factor is expected to exert a net effect across other factors, and subsequently to affect the overall depressive symptomology. Of note, there are more psychological (e.g., cognitive biases) and biological factors (e.g., serotonin transporter genotype) that are known to be involved in depression (36, 37); this review is limited to the six selected factors.

The sum of deficits within these factors has been shown to result in profound impairments in daily functioning (38), a reduced quality of life (38, 39), an increased risk of suicide (40), and a substantial lack of productivity (41, 42). This also renders MDD costly on an economical level. Estimates of the financial burden that can be ascribed to occupational incapacity due to depression approximate 33 billion euro per year in the United States of America alone, excluding treatment costs (43). Taken together it is clear that there is a pressing need to come up with alternative treatments for depression, next to the conventional first-line psycho- and pharmaco-therapies.

### Conventional Treatments of Depression

A wide array of biological (“pharmacotherapy”) and psychological (“psychotherapy”) treatment options for depression is currently available, targeting different elements that are thought to be the underlying pathological cause in their specific theoretical framework (3). Common pharmacotherapy is predominantly based on the hypothesis that depression is caused by a deficiency of monoamine neurotransmitters, such as serotonin (5-HT), dopamine, and norepinephrine, and their receptors, which play an important role in the regulation of mood, arousal, and memory. By elevating these neurotransmitter levels to varying degrees, different types of antidepressants, such as selective serotonin reuptake inhibitors (SSRIs) or monoamine-oxidase inhibitors, are assumed to reduce depressive symptoms (44). However, while being only partially effective in severe cases of depression, antidepressants (45) may also cause severe adverse effects, such as sexual dysfunction or cardiovascular risks (46, 47). Moreover, upon discontinuation, relapse rates are high, which is why antidepressants are often taken chronically (48).

Psychotherapy includes cognitive-behavioral therapy (CBT) and interpersonal therapy, both based on different psychosocial theories, focused on modifying, respectively, behavioral and cognitive biases by means of repeated counseling sessions with a therapist (3). Despite large effects in reducing depressive symptoms (49), relapse and drop-out rates are considerably high (50, 51). For this reason, common pharmaco- and psychotherapies for MDD are frequently combined, which has been acknowledged to be more effective than either approach alone (52). Nevertheless, a substantial proportion of MDD patients that fails to achieve full recovery remains, with almost 75% after 8 weeks and approximately a quarter after 24 weeks of treatment (53).

### Alternative Treatments of Depression

In response to the profound limitations of conventional treatments of depression, several alternatives have been proposed (e.g., 54–56). Among these, two approaches that originate from spiritual practice traditions of indigenous and religious communities, namely mindfulness meditation (MM) and administration of classical psychedelics, have gained scientific interest in depression research (57, 58). With regard to the latter, a limited number of clinical trials have been conducted in depressed patients who were administered ayahuasca and psilocybin (59). Although these studies give preliminary evidence of their potential in the treatment of depression, caution regarding efficacy conclusions in depression is warranted due to the currently limited number of studies and small sample sizes (60–63).

For this review we have chosen to focus on psilocybin given the known safety profile in individuals (64) and the potential future of psilocybin therapy since it has received the “breakthrough therapy for treatment-resistant depression (TRD)” designation from the FDA (October 2018). The latter means that the FDA acknowledges that “there is preliminary clinical evidence that indicates that the drug may demonstrate substantial improvement over existing therapies on one or more clinically significant endpoints, such as substantial treatment effects observed early in clinical development and that it will be used to treat a serious or life threatening disease or condition” (65).

MM and psilocybin are thought to exert their effects on behavior *via* a variety of psychological and biological mechanisms, potentially resulting in expeditious and long-lasting effects (e.g., 66–69).

#### Mindfulness Meditation

MM is a form of meditation derived from the Pali word “sati” that emphasizes the mental practice of present moment awareness in a non-judgemental and emotionally accepting fashion while remaining in a relaxed state (70, 71). In healthy populations, protracted MM practice (of several months) is linked to improvements in self-regulation and subjective well-being (72, 73). Of note, also shorter MM training (of e.g., four days) already has a positive impact on mood and executive functioning, while reducing fatigue and anxiety (74).

Different forms of MM may be applied, depending on the meditator’s expertise and personal goals. Focused-attention meditation (FAM) involves the direction of attention towards a focal object and gentle reinstatement of this focus when thoughts drift off or strong emotions surface (75). This variant is usually employed by novice meditators. Open-monitoring meditation (OMM) involves no focal object, but rather non-selective awareness of the present moment, and is preferably operationalized among more advanced meditators. Loving-kindness meditation (LKM), on the other hand, combines technical components of FAM and OMM, and puts strong emphasis on the fostering of compassion and positive emotions (76, 77).

MM can be used as a supplement in psychotherapy, constituting mindfulness-based interventions (MBIs), of which mindfulness-based stress reduction (MBSR) and mindfulness-based cognitive therapy (MBCT) are the most common (78). These usually entail sessions guided by a professional in addition to at-home practice over a duration of eight weeks (78). MBSR specifically targets the management of stressful situations and is recommended as a supportive means in chronic diseases, whereas MBCT teaches strategies for dealing with maladaptive thought patterns, which makes it more suitable for the prevention of depressive relapse (60, 79).

Not only have MBIs demonstrated efficacy in the treatment of depression (61), but they are also effective in reducing symptoms in a variety of other psychiatric and medical conditions, such as social anxiety, drug-resistant epilepsy, and mental fatigue following brain damage (80–82). Due to its particularly enduring effects, MM is frequently incorporated as an adjunct in maintenance treatments for the prevention of relapse of depressive symptoms (83, 84) or utilized as alternative treatment in treatment-resistant patients (61). However, effect sizes of MBIs are only moderate (85), and, in order to fully benefit from mindfulness training, a certain meditation depth is required, which depends on individual predispositions and practice frequency (86).

#### Psilocybin

Psilocybin (4-phosphoryloxy-N,N-dimethyltryptamine), on the other hand, is a classical tryptamine hallucinogen that can be derived from a variety of Psilocybe mushroom species (87). Upon oral administration, subjective effects become apparent after approximately 30 to 60 min, peak 90 to 180 min later and last up to 6 h in total (88). These effects are highly dose-dependent (89) and entail perceptual, cognitive, and emotional alterations, which may resemble the features of psychosis (90).

As psilocybin is metabolized into psilocin (4-hydroxy-N,N-dimethyltryptamine) upon ingestion, it is regarded as a prodrug (87, 91). Psilocin acts as a 5-HT agonist and has a particularly high affinity for the 5-HT2A receptor subtype, which is thought to be responsible for its psychotropic effects (92). An analysis by Johnson et al. (64) shows that, although psilocybin has some level of abuse potential and risk, there is no strong evidence of physical dependence, and it can in general be safely used under medical supervision. Nonetheless, adverse effects, such as anxiety or psychotic reactions, may occur when psilocybin is administered in an environment that could evoke negative emotions, as the drug tends to amplify the present affective state (93). Thus, the provision of psychological support and surroundings that are perceived as comfortable and safe are essential when applying psilocybin or other psychedelics in empirical or clinical trials (94).

If these precautions are taken, psilocybin suggests to promote long-lasting positive changes in well-being, attitude, and personality upon a single administration (88, 95). Apart from its potential therapeutic value in depression (63, 96, 97), psilocybin also holds promise for a variety of other conditions, such as anxiety in terminal illness, obsessive–compulsive disorder, and substance dependence (98–100). However, this evidence is still largely based on a limited number of small-scale controlled studies and hence preliminary, impeding its approval for clinical practice.

## Aim and Outline

Due to its intrinsically mental and observant nature, MM can be seen as a psychologically focused approach; it is a specific way of paying attention, with a focus on being in the present, in a non-judgemental way. Similar to other trainings or exercises, MM has effects on neurobiological processes (101). Psilocybin is a pharmacological agent, acutely affecting neurobiological processes and inducing psychological effects. Both MM and psilocybin induce structural—longer-lasting—psychological and biological changes, and they show potential of being valuable novel alternatives in the treatment of depression.

In clinical psychedelic patient trials, psilocybin is always administered in a supportive setting, and followed by multiple integration sessions after the experience. Here, the inclusion of MM in the psychedelic-assisted psychotherapy might yield larger or longer positive effects than either treatment on its own, similar to the conjunction of conventional pharmacotherapy and psychotherapy (52).

Noteworthy here is that meditative elements, such as inward-directed attention and relaxation practices, are already being incorporated in psilocybin-assisted trials (69, 102, 103). One study in healthy volunteers concluded that determinants of the longer-lasting positive effects on prosocial attitudes and behaviors as well as psychological functioning following psilocybin administration were the psilocybin-occasioned mystical-type experience and the rate of meditative or spiritual practices (69). While very interesting and relevant in light of current developments psychedelic research, lacking here is a firm theoretical ground of such implications, as none of those studies has directly tested potential applications of a combination of both MM and psilocybin in the treatment of depression (69, 104).

Therefore, the present review aims to compare acute and long-term effects of MM and psilocybin on psychological and biological factors associated with depression in order to provide a theoretical understanding of the potential benefit when used in combination in the treatment of MDD. In each section, findings on the effects of MM and psilocybin will be presented with respect to the aforementioned six factors as depicted in **Figure 1**, followed by inferences about the potential beneficial effects when using them in combination.

The effects of MM and psilocybin are considered additive or complementary when their comparison suggests they both exert positive effects on the same factor, and when a theoretical combination is reasonably likely to yield superior effects over either treatment individually, with regard to effect duration (i.e., acute versus long-term effect), and/or underlying working mechanism (e.g., bottom-up versus top-down effect).

## Methods

In order to gain information on the individual effects and underlying working mechanisms of MM and psilocybin on the six selected MDD-related factors (three psychological, three biological), empirical articles, textbooks, and review papers were searched between September 2018 and January 2019, using the databases PubMed and Google Scholar. Three separate search strings were employed for “depression,” “mindfulness meditation,” and “psilocybin,” which were, combined with the Boolean command “OR.”

The search string for “depression” included terms related to the six factors proposed in the introduction (i.e., depression, mood, cognitive, social, interpersonal, neuroplasticity, BDNF, network, HPA axis, cortisol, stress, inflammation). The search string for “mindfulness meditation” included MM-associated concepts and interventions (i.e., mindfulness, meditation, mindfulness-based intervention, mindfulness-based cognitive therapy, mindfulness-based stress reduction). Although the focus of the review was on psilocybin, the search string for “psilocybin” also contained other classical psychedelics, as similar mechanisms of action may allow for inferences about psilocybin’s potential effects (i.e., psilocybin, psilocin, psilocybin-assisted therapy, tryptamine, LSD, ayahuasca, DMT, 5-HT2A, psychedelic, hallucinogen).

**Figure 2:**
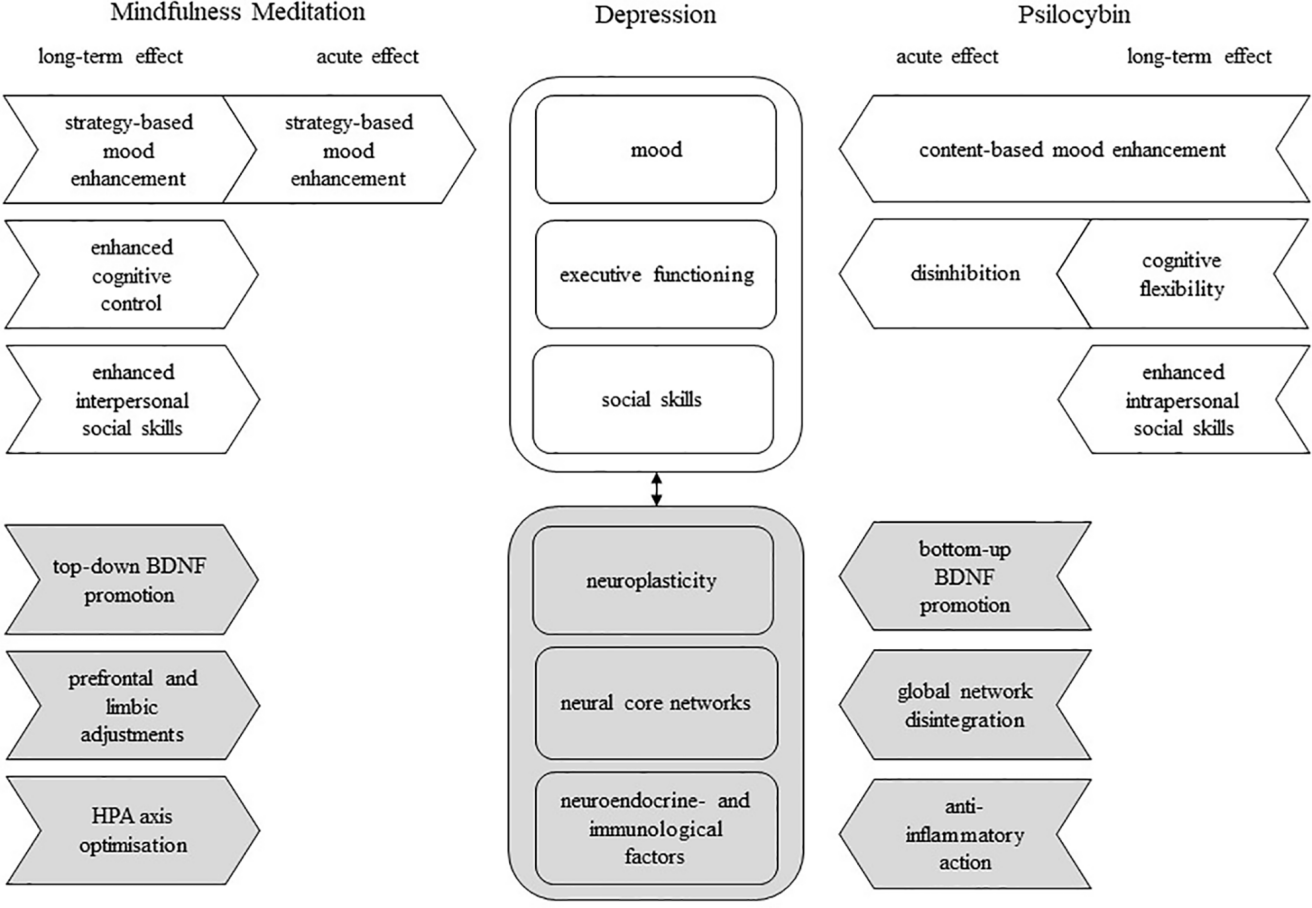
A model of possible complementary effects of mindfulness meditation (MM) and psilocybin on psychological and biological deficiencies associated with major depressive disorder; rounded square-shaped box, deficient factor(s) in depression; arrow-shaped box, unidirectional effect; white box, psychological factor/effect; gray box, biological factor/effect; black arrow, interdependence; BDNF, brain-derived neurotrophic factor.

An article was included when its focus was on psilocybin (or a classical psychedelic) and/or mindfulness, and comprised information relevant to the treatment of MDD. In addition, reference lists of included articles were searched. The literature search yielded a total of 1129 hits, of which 1047 publications were excluded since they did not match the inclusion criteria based on their title or abstract. The remaining 82 articles were individually analyzed and assigned to one or more of the six factors associated with depression; 13 articles were added after reference search lists of the included articles. In total, 95 articles were used for the present review (**Figure 2**). Among those, 67 were papers describing original (“experimental”) research, seven were original research articles without an experimental manipulation (correlational), three were pooled data analyses, 12 were review papers, and the remaining six included theoretical (3) or editorial (1) pieces and one book. A summary of the papers describing original research is presented in **Table 1**.

**Table 1:** Overview of experimental studies and other types of studies (e.g., no intervention or pooled data analysis) included in the review; only constructs and findings (increase ↑/reduction ↓) related to the model are presented; *the most used abbreviations are BS, Between Subject; WS, Within Subject; MM, Mindfulness Meditation (or related); P, Psychedelic; Psi, Psilocybin; p.o., per oral; IV, intravenously; the other abbreviations are explained in the footnote of this table*.

Study	Sample (size)	Design/Intervention	Construct (measure)	Findings	MM/P
Astin (105)	Healthy participants (N=28)	BS, 2 groups: 8-weekBSR programme or non-intervention control condition	Psychological symptomology (SCL-90; perceived control; spiritual experience)	MBSR group > control group: ↓ overall psychological symptomatology, ↑ overall domain-specific sense of control, ↑ scores on a measure of spiritual experiences	MM
Alonso et al. (106)	Healthy male volunteers with prior experience with psychedelics (N=10)	WS, 2 sessions: placebo and freeze-dried ayahuasca (0.75 mg DMT/kg equivalent dose, p.o.)	Directed functional connectivity (transfer entropy)	Ayahuasca: ↓ top-down control (anterior regions), ↑ bottom-up (posterior regions) information transfer in the human brain	P
Barnes et al. (107)	Study 1: dating undergraduate students (N=89); Study 2: 30 heterosexual couples (N=60)	Study 1 (WS): short-term longitudinal measurement over 10 weeks; Study 2 (WS): Conflict discussion paradigm	Both studies: Mindfulness (MAAS); Relationship satisfaction (DAS; IMS); Self-control (SCS); Only study 2: Mood (POMS); communicative and affective functioning (SCID); changes in perception of the partner and relationship	Mindfulness: ↑ relationship satisfaction, ↑ capacities to respond constructively to relationship stress, ↓ emotional stress responses, positive pre- and post-conflict change in perception of the relationship, ↑communication quality	MM
Barrett et al. (93)	Healthy hallucinogen users (N=20)	Double-blind, WS, 5 sessions: Psi (10, 20, and 30 mg/70 kg, p.o.), DXM (400 mg/70 kg, p.o.) and placebo	Psychomotor performance (motor praxis task); Memory (word-encoding, recall, and recognition task; letter N-back task); Visual perception (PLOT)	Psi: ↓ psychomotor performance, WM, episodic memory, associative learning, visual perception; dose-dependent effects	P
Broderick (108)	Healthy undergraduate students (N=177)	BS; negative mood induction + rumination (group 1), distraction (group 2), or MM (group 3)	Affect (PANAS); Thought-listing	Negative affect: group 3 < group 1 and 2	MM
Brown et al. (109)	Healthy undergraduate students (N=44)	BS; Trier Social Stress Test or control task	Cortisol; Mindfulness (MAAS); Perceived stress (PSS), Anxiety (POMS); Negative affectivity (PANAS); Fear of negative evaluation (FNE)	Mindfulness: ↓ cortisol response to social stress, anxiety, negative affect	MM
Cahn et al. (110)	Healthy participants (N=38)	WS; 3-month yoga and meditation retreat	Psychometric measures (BSI, FMI, Tellegen Absorption Scale), serum BDNF levels; circadian salivary cortisol levels; pro- and anti-inflammatory cytokines	↓ Anxiety, depression, pro-inflammatory cytokine IL-12; ↑ mindfulness, BDNF, CAR), anti-inflammatory cytokine IL-10 plasma levels, other pro-inflammatory cytokines	MM
Barrett et al. (93)	Healthy participants, not drug naive (N=20)	Double-blind, WS, 5 sessions: Psi (10, 20, 30 mg/70 kg, p.o.), DXM (400 mg/70 kg, p.o.), and placebo	Subjective drug effects (Subjective Effects Questionnaire, Drug effect intensity rating, SOCQ, 5D-ASC, Mysticism Scale, Psychological Insight Questionnaire, Challenging Experience Questionnaire)	Psi (30 mg/kg) and DXM (400 mg/70 kg): similar profiles of subjective experiences ↓ psychomotor performance, balance; Psi > DXM: visual, mystical-type, insightful, and musical experiences; Psi < DXM: disembodiment, nausea/emesis, light-headedness	P
Carhart-Harris et al. (111)	Healthy participants with previous experience with a hallucinogenic drug (N=30)	WS, 2 sessions: Psi (2 mg in 10-mL saline) and placebo	CBF (fMRI); changes in venous oxygenation; intensity of subjective effects	Psi: ↓ CBF, which was maximal in hub regions (e.g. thalamus, ACC, PCC),↓ positive coupling between mPFC and PCC., ↓ ACC activity predicted the intensity of the subjective effects	P
Carhart-Harris et al. (112)	Healthy participants (N=15)	WS, 2 session: Psi (2 mg, IV + 10 mL saline) and placebo (10 mL saline)	Functional connectivity (fMRI)	Psi: ↑ functional connectivity between DMN and CEN, preserved thalamocortical connectivity	P
Carhart-Harris et al. (103)	Unipolar, treatment-resistant major depression patients (moderate-to-severe) (N=12)	Open-label, two doses of Psi (10 mg and 25 mg, p.o.) 7 days apart, supportive setting	Depressive symptoms (QIDS, BDI, HAM-D, MADRS, GAF), Anxiety (STAI), Anhedonia (SHAPS)	↓ Depressive symptoms, anxiety, anhedonia 1 week and 3 months after (25 mg) treatment compared to baseline	P
Carhart-Harris et al. (63)	Patients with moderate-to-severe, unipolar, treatment-resistant major depression (N=20)	Open-label, uncontrolled administration of Psi (10 mg and 25 mg, p.o. 7 days apart), supportive setting	Depressive symptoms (QIDS), BDI, HAM-D, MADRS, GAF), state-trait anxiety (STAI), anhedonia (SHAPS)	↓ Depressive symptoms until 6-month follow-up; response (n=9) was predicted by acute psychedelic experience quality; remitters (n=4)	P
Carlson et al. (113)	Early stage breast and prostate cancer patients (N=42)	8-week MBSR programme	QOL (EORTC QLQ-C30); Mood (POMS), stress symptoms (SOSI); Salivary cortisol; Plasma DHEAS; Salivary melatonin	↑ QOL, associated with ↓ afternoon cortisol levels; ↑ sleep quality; ↓ symptoms of stress;	MM
Carlson et al. (114)	Cancer outpatients (N=54)	7-week MM programme	Mood (POMS); Stress symptoms (SOSI)	↓ Mood disturbance and stress until 6-month follow-up	MM
Carter et al. (115)	Healthy participants (N=8)	Double-blind, WS, 4 sessions: placebo, ketanserin (50 mg, p.o.), Psi (215 mg/kg, p.o.), and Psi + ketanserin	Attentional tracking ability (multiple-object tracking task); Spatial WM (spatial WM task)	Psi: ↓ attentional tracking ability (not attenuated by pre-treatment with ketanserin)	P
Carter et al. (116)	Healthy participants (N=10)	Double-blind, WS, 4 sessions: pre-treatment with ketanserin (50 mg, p.o.) or placebo, followed by administration of Psi (215 µg/kg, p.o.) or placebo	Subjective drug effects (AMRS, 5D-ASC), Binocular rivalry (binocular rivalry switch rate)	Psi: ↓ rate of binocular rivalry switching (not blocked by ketanserin); ↑ proportion of transitional/mixed percept experience; ketanserin: ↓ P’s hallucinogenic symptoms	P
Coffey and Hartman (117)	2 independent, healthy undergraduate student samples (sample 1: n = 204; sample 2: n = 258)	Observational study; Structural equation modeling to test the mediation role of emotion regulation, nonattachment, and reduced rumination in in the relationship between mindfulness and a psychological distress factor	Mindfulness (MAAS); Emotion regulation (TMMS-Repair subscale); Nonattachment (Linking Inventory); Rumination (RRQ); Psychological distress (BSI)	Inverse relationship between mindfulness and psychological distress and emotion regulation, nonattachment, and rumination significantly mediated this relationship.	MM
Domínguez-Clavé et al. (118)	Volunteers (N= 45; of which 12 with borderline personality disorder (BPD) traits)	Naturalistic ayahuasca study; tests were administered prior to and 24 h after the ayahuasca session	Emotion regulation (DERS); mindfulness (FFMQ), Decentering (EQ); Borderline Personality Disorder (MSI-BPD)	Participants ↑ in mindfulness capacities and emotion regulation. The BPD-like subgroup ↑in emotion regulation; no changes in mindfulness capacities	P
Frecska et al. (119)	Participants of ayahuasca rituals (N= 40)	Naturalistic study; Assessments before and two days after the end of a two-week long ceremony	Visual creativity (Torrance Tests of Creative Thinking)	Highly original solutions and phosphenic responses ↑ number	P
Gex-Fabry et al. (120)	Patients remitted from recurrent depression (N = 56; MBCT: n = 28; TAU: n = 28)	BS: 8-week MBCT + TAU or TAU alone	Cortisol (CAR, average day exposure, diurnal slope); depression (MADRS, BDI); relapse (SCID)	Unchanged cortisol secretion patterns following participation in MBCT or TAU	MM
Gotink et al. (121)	Adult participants (previously completed MBSR or MBCT course) (N=29)	WS+BS: Mindful walking in nature for 1, 3, 6, or 10 days with a control period of a similar number a of days 1 week before the mindful walking period	Affect and state mindfulness (ESM); Depression, anxiety and stress (DASS-21); Mindfulness skills (Toronto Mindfulness Scale)	Mindful walking: ↑ mindfulness (predicted ↓ negative affect), positive affect, state mindfulness; positive affect and mindfulness prospectively enhanced each other in an upward spiral	MM
Greenberg et al. (122)	Study 1 (N=35): experienced mindfulness meditators (n=14) and non-meditators registered for their first meditation retreat (n=21); Study 2 (N=76): non-meditators	Study 1: BS; Study 2: BS, 8-meeting mindfulness programme or waiting list	Cognitive rigidity (Einstellung water jar task (Study 1 and 2)), Alphabet-Maze task (Study 2)	Rigidity scores experienced mindfulness meditators < non-meditators; rigidity scores non-meditators with mindfulness programme < waiting list group	MM
Greenberg et al. (123)	Non-meditators (N=76)	BS; 8-session mindfulness training programme or waiting list	Task set inhibition (BI, CRS)	Mindfulness programme: ↑ BI, but not CRS, compared to a waiting list group	MM
Griffiths et al. (88)	Healthy participants (N=18)	Double-blind, WS, 5 sessions: Psi (5, 10, 20, 30 mg/70 kg, p.o.) and placebo	acute subjective drug effects (HRS, APZ, 5D-ASC, Mysticism Scale, SOCQ, ARCI); retrospective persisting effects (PEQ); attitudes/dispositions/behavior (Mysticism Scale, Death Transcendence Scale, Community Observer Ratings)	Psi (20 and 30 mg/70 kg): mystical-type experiences that have persisting (1 and 14 months) positive effects (↑) on attitudes, mood, and behavior	P
Griffiths et al. (96)	Cancer patients with life-threatening diagnoses and symptoms of depression and/or anxiety (N=51)	Randomized, double-blind, WS: Psi (1 or 3 mg/70 kg, p.o.) and Psi (22 or 30 mg/70 kg, p.o.)	Depression (HAM-D, BDI, HADS); Anxiety (HAM-A, HADS, STAI, LAP); Mood (POMS); Self-rated psychiatric symptoms (BSI); Wellbeing (QOL); Optimism (LOT-R); Life meaningfulness (PILT); Understanding of self/others/life (LAP); Other-ratings of participant’s mood/attitude/behavior	Psi (22 or 30 mg/70 kg): ↓ depressed mood, anxiety, ↑ QOL, life meaning, optimism, up until 6-months; participants attributed ↑ in attitudes to Psi (20 or 30 mg/70 kg) experience, consistent with other-ratings; mystical-type Psi experience on session day mediated the effect of Psi dose on therapeutic outcomes.	P
Griffiths et al. (69)	Healthy participants (N=75; 25 per condition)	BS; 6-8-month programme of meditation/spiritual practices + double-blind administration of one of three doses of Psi p.o.: (1) 1 mg/70 kg + moderate-level support; (2) 20 and 30 mg/70 kg + moderate-level support; (3) 20 and 30 mg/70kg + high support	Persisting effects attitudes (Brief RCOPE, Gratitude Questionnaire, LAP, Trait Forgiveness Scale, Inclusion of Others in the Self scale, ASPIRES, Dispositional Positive Emotions Scale, LOT-R, SWLS, PILT, Other-ratings of participant’s mood/attitude/behavior	Psi: ↑ interpersonal closeness, gratitude, life meaning/purpose, forgiveness, death transcendence, daily spiritual experiences, religious faith and coping, community observer ratings at 6 months; Psi (20 and 30 mg/kg) > Psi 1 mg/kg; enduring effects depended on Psi-occasioned mystical-type experience and rates of meditation/spiritual practices	MM+P
Griffiths et al. (124)	Healthy adults who regularly participate in religious/spiritual activities (N=36), hallucinogenic-naïve	WS, 2-3 sessions: Psi (30 mg/70 kg, p.o.) and methylphenidate (40 mg/70 kg, p.o.)	Affect (PANAS); Well-being (QOL); personality (NEO-PI); spirituality (Spiritual Transcendence Scale; Faith Maturity Scale; FACIT)	Psi: ↑ positive attitudes, mood, social effects and behavior at 2- and 14-month follow-up; Psi occasioned experiences similar to spontaneously occurring mystical experiences	P
Griffiths et al. (102)	Hallucinogen-naïve healthy adults who regularly participate in religious/spiritual activities (N= 36)	WS, 2-3 sessions: Psi (30 mg/70 kg, p.o.) and methylphenidate (40 mg/70 kg, p.o.)	Affect (PANAS); Spirituality (Mysticism scale); Other-ratings of participant’s mood/attitude/behavior	Psi: 2 months self- and other-rated sustained positive changes in attitudes and behavior	P
Grob et al. (99)	Adults with advanced-stage cancer, and anxiety (N=12)	Double-blind, WS, placebo-controlled, Psi (0.2 mg/kg, p.o.)	Depression (BDI); Mood (POMS); Anxiety (STAI); Psychiatric symptoms (BPRS)	Psi: ↓ anxiety, depressive symptoms at 1 and 3 months	P
Håkansson et al. (125)	Healthy older participants (N=19)	WS, 3 sessions: 35 min of physical exercise (condition 1), cognitive training (condition 2) or mindfulness practice (condition 3)	Neuroplasticity (serum BDNF levels)	Condition 1 > condition 2-3: ↑ serum BDNF levels	MM
Hargus et al. (126)	Depressed patients who had experienced suicidal crises (N=27)	BS; MBCT + TAU or TAU alone	Depression (SCID, BDI); meta-awareness (MACAM); specificity of memory (ReSSI)	MBCT > TAU: ↑ meta-awareness, specificity of memory	MM
Harman et al. (127)	Professionally employed males (N=27)	WS: mescaline (200 mg, p.o.)	Creativity (Purdue Creativity Test, Miller Object Visualization Test, Witkin Embedded Figures Test); Subjective ratings	↑ creative problem-solving for at least some weeks subsequent to the psychedelic session	P
Hasler et al. (128)	Healthy participants (N=8)	Double-blind, WS, 5 sessions: placebo, Psi (45, 115, 215 and 315 μg/kg, p.o.)	Attention (FAIR); Mood (AMRS), blood chemistry (plasma concentrations of thyroid-stimulating hormone, prolactin, cortisol, adrenocorticotropic hormone)	Psi: ↑ physiological parameters in a dose-dependent manner; Psi (215 & 315 μg/kg): ↓ attentional capacity	P
Hölzel et al. (129)	Healthy participants (N=26)	WS; 8-week MBSR programme	Perceived stress (PSS); amygdala grey matter density (MRI)	↓ stress, correlated with ↓ right basolateral amygdala grey matter density	MM
House et al. (130)	Mouse cells	In vitro treatment with LSD (100 µm)	Inflammatory markers	↓ proliferation of B-lymphocytes, production of the cytokines IL-2/IL-4/IL-6, induction of cytotoxic T-lymphocytes, NK response, ↑ basal and IL-2-augmented NK cell function	P
Huffziger and Kuehner (131)	Depressed patients 3.5 years after discharge from inpatient treatment (N=76)	BS; Negative mood induction + rumination (group 1), distraction (group 2), or mindful self-focus (group 3)	Depression (SCID-I); Coping styles (RSQ); Mindfulness (FMI); Mood (PANAS)	Negative mood group 2-3 < group 1; High mindfulness facilitates negative mood reduction in group 3	MM
Jha et al. (132)	Experimental mindfulness-naive participants (group 1; n=17), experienced meditators (group 2 n=17) and a mindfulness-naive control group (group 3; n=17)	BS; group 1 participated in an 8-week MBSR course, while group 2 participated in a 1-month intensive mindfulness retreat	Alerting/orienting/conflict monitoring (ANT)	Pretraining: group 2 > 1 conflict monitoring; post-training: group 1 ↑ endogenous orienting; group 2 ↑ exogenous alerting	MM
Jimenez et al. (133)	College students (N = 514)	Observational, cross-sectional study; Structural equation modeling to test the relationship between self-rated dispositional mindfulness and depressive symptoms through emotion regulation, mood regulation and self-regulation	Positive emotions (mDES), Perceived mood repair ability (NMR-15); Self-acceptance (PWBS); Mindfulness (FMI); Depressive symptoms (CES-D)	Relationship between mindfulness and depressive symptoms was mediated by emotion regulation, mood regulation and self-regulation. Higher levels of dispositional mindfulness were associated with higher levels of positive emotions, mood regulation expectancies, and self-acceptance; all were inversely related to depressive symptoms. Self-acceptance was the strongest mediator of mindfulness and depressive symptoms.	MM
Kometer et al. (134)	Healthy participants (N=17)	Randomized, double-blind, 4 sessions: Psi (215 g/kg), ketanserin (50 mg), Psi + ketanserin, placebo	Affect (PANAS), Anxiety (STAI-State), Emotion recognition (FERT); Emotional Go/NoGo Task, EEG	Psi: ↑ positive mood, goal-directed behavior (++ > – cues), positive emotion; ↓ recognition of negative facial expression (blocked by ketanserin), negative sequential emotion, P300 component (valence-dependent)	P
Kraehenmann et al. (135)	Healthy participants (N=25)	Double-blind, WS, 2 sessions: Psi (0.16 mg/kg, p.o.) and placebo	Affect (PANAS) Anxiety (STAI); Amygdala reactivity (amygdala reactivity task; BOLD fMRI)	Amygdala reactivity to negative and neutral stimuli Psi < placebo; this response (after Psi) was related to the Psi-induced ↑ positive affect	P
Kubera et al. (136)	Human blood samples from healthy volunteers (N=19) and major depressed patients with treatment resistant depression (n= 7; N=26)	Treatment with 5-HT (150, 1.5 and 15 μg/mL), PCPA (5 μM), flesinoxan (15 and 1.5 μg/mL), mCPP (27 and 2.7 μg/mL), and ritanserin (50 and 5.0 μg/mL)	Cytokine secretion (LPS, PHA); pro- versus anti-inflammatory capacity of cultured whole blood (IFNγ/IL-10 production ratio)	5-HT: ↓ IFNγ/IL-10 ratio; PCPA: ↓ production of IFNγ and IL-10; mCPP and ritanserin: ↓ IFNγ/IL-10 ratio	P
Kuypers et al. (137)	Healthy participants (N=26)	Naturalistic ayahuasca study; Assessment took place at baseline and 2 h after drinking ayahuasca	Divergent thinking ((PLMT), Convergent thinking (PCT)	Convergent thinking ↓, divergent thinking ↑	P
Lazar et al. (138)	Healthy participants with extensive Insight meditation experience (N= 20) and control participants with no meditation or yoga experience (N= 15)	BS Structural MRI; scanning	Cortical thickness (MRI)	Brain regions associated with attention, interoception and sensory processing were thicker in meditation participants than controls, including the PFC and right anterior insula.	MM
Lee et al. (139)	Male Chinese expert meditators N(FAM)= 11; N(LKM)= 11; male Chinese novice meditators N(FAM)= 11; N(LKM)=11	Cognitive tasks during fMRI scanning	Cognitive processing (CPT); Affective processing (EPT); brain activity (BOLD fMRI); affect (CAS)	FAM was associated with expertise-related behavioral improvements and neural activation differences in attention task performance, in contrast to LKM meditation. Both FAM and LKM practice affected the neural responses to affective pictures: For viewing sad faces, the regions activated for FAM experts were consistent with attention-related processing, whereas responses of LKM experts to sad pictures were more in line with differentiating emotional contagion from compassion/emotional regulation processes.	MM
Lengacher et al. (140)	Advanced-stage cancer patients (n=26) and caregivers (n=26; N=52)	BS+WS; modified 6-week, self-study MBSR-C	Perceived stress (PSS); Depression (CES-D); Anxiety (STAI); Psychological and physical symptoms (MSAS); QOL (MOS); Stress markers (salivary cortisol and IL-6)	Patients and caregivers: ↓ cortisol at weeks 1 and 3, ↓ IL-6 levels at week 6; patients: ↓ stress, anxiety; caregivers: ↓ psychological symptoms, ↑ QOL (not significant);	MM
Lewis et al. (141)	Healthy participants (N=58)	Double-blind, BS (Psi dose)+WS, 2 sessions: group 1: 0.160 mg/kg Psi p.o.; group 2: 0.215 mg/kg Psi p.o., and placebo	perfusion changes (fMRI); subjective drug effects (5D-ASC)	Group 1 < 2 ↑ subjective drug effects; Psi: ↑ relative perfusion in right hemispheric frontal and temporal regions and bilaterally in the anterior insula, ↓ relative perfusion in left hemispheric parietal and temporal cortices subcortical regions, widespread ↓ in absolute perfusion	P
Lutz et al. (142)	Healthy participants (experimental: n=24; control: n=22)	BS: short mindfulness intervention, followed by emotional expectation paradigm during fMRI scanning	Psychometric measures (SDS; STAI; Eysenck Personality Inventory; EPI; FMI; MAAS) Brain activity (fMRI);	Mindfulness intervention: ↑ activations in prefrontal regions during expectation of (potentially) negative stimuli, ↓ activation in amygdala and parahippocampal gyrus during perception of negative stimuli; prefrontal and right insular activations when expecting negative pictures correlated negatively with trait mindfulness	MM
Ly et al. (143)	In vitro: cultured cortical rat neurons; *in vivo*: Drosophila larvae, zebrafish embryos	In vitro studies: treatment of cultured cortical rat cells with different psychedelic class compounds (amphetamines, ergolines, tryptamines, iboga); *in vivo* studies: administration of DMT or ketamine (10 mg/kg)	Structural change (fluorescence microscopy); Functional change (electrophysiology); Neuroplasticity (BDNF levels)	Serotonergic psychedelics: ↑ neuritogenesis, spinogenesis, synapse number and function; induced structural changes appear to result from stimulation of the TrkB, mTOR, and 5-HT2A signaling pathways.	P
MacLean et al. (144)	Healthy, hallucinogen-naïve participants (N= 52)	Pooled data analysis; Combination of Psi data from Griffiths et al. (102) and Griffiths et al. (82)	Personality (NEO-PI)	Psi: ↑ Openness; In participants who had mystical experiences during their psilocybin session, Openness remained significantly higher than baseline more than 1 year after the session.	P
Malarkey et al. (145)	University faculty and staff (n=186) with elevated CRP level (> 3.0 mg/ml) who had, or were at risk for cardiovascular disease	BS; 2-month MBI-ld (group 1) or lifestyle education programme (group 2)	CRP; IL-6; cortisol; Perceived stress (PSS); Depression (CES-D); Sleep quality (PSQI)	Group 1 > 2: ↑ mindfulness at 2-months and up to a year	MM
Mason et al. (95)	Healthy participants (before ingesting P N=55; the morning after P: N=50); 7 days after P: N=22)	WS; ingestion of truffles (1.9 mg, p.o.) containing Psi in a retreat setting	Creative thinking (PCT), empathy (MET), Satisfaction with life (SWLS)	Psi ↑ divergent thinking & emotional empathy the morning after use; 7 days after Psi: ↑ convergent thinking, valence-specific emotional empathy, and well-being; changes in empathy correlated with changes in wellbeing	P
Matousek et al. (146)	Women with breast cancer and depressive symptoms (N=33)	WS; MBSR programme	Stress (CAR; PSS); Depression (CES-D); medical symptoms (MSCL)	Prolonged ↑ CAR, ↓ self-reported stress levels, depressive symptomatology, medical symptoms	MM
Moore and Malinowksi, (147)	Experienced Buddhist meditators (N= 25) and a meditation-naïve control group (N= 25)	Observational study	Mindfulness (KIMS); degree of automatization/deautomatization (Stroop task); Attentional performance and flexibility (d2-concentration and endurance test)	Attentional performance and cognitive flexibility were positively related to meditation practice and levels of mindfulness	MM
Mrazek et al. (148)	Healthy undergraduate students (N=48)	WS; 2-week mindfulness-training course	WM capacity (OSPAN); Mind wandering (Thought sampling); Reading comprehension (GRE)	Mindfulness training: ↑ reading comprehension; WM capacity, ↓ mind wandering; improvements in performance were mediated by reduced mind wandering among participants prone to distraction at pretesting	MM
Nau et al. (149)	Young adult male mice	BS; sterile saline or (R)-DOI, followed by TNF-α; pretreatment with a 5-HT2A receptor antagonist. in some mice	Gene expression; protein expression; cytokines	(R)-DOI: ↓ inflammation by blocking the systemic effects of TNF-α (blocked by pre-treatment with a 5-HT2A receptors antagonist)	P
Oken et al. (150)	Community-dwelling caregivers of close relatives with dementia (N=28)	BS, 7 weeks: MBCT-based programme (group 1), education class based on Powerful Tools for Caregivers (group 2) or respite	Stress (RMBPC); mood (CES-D); fatigue (SF-36); self-efficacy (General Perceived Self-Efficacy Scale); mindfulness (MAAS, FFNJ); cortisol; cytokines; cognitive function (10-word list learning task, Stroop task, ANT); expectancy of improvement; credibility of the interventions	Group 1 and 2: ↓ stress, ↑ self-efficacy, cognitive function; significant correlations between mindfulness and self-rated mood and stress scores	MM
Pokorny et al. (151)	Healthy participants (N=32; N(MET)=32; N(MDT)= 24)	Double-blind, WS, 2 sessions: Psi (0.215 mg/kg, p.o), placebo	Empathy (MET, IRI); Moral decision-making (MDT); Affect (PANAS)	Psi > placebo: ↑ emotional empathy	P
Preller et al. (152)	Healthy participants (N=21)	Double-blind, WS, 2 sessions: Psi (0.215 mg/kg, p.o.) and placebo, followed by exposure to social ostracism	Neural activity (fMRI); Empathy (MET)	Psi: ↓ ACC response to social ostracism, ↑ emotional empathy compared to placebo	P
Quednow et al. (153)	Healthy participants (N=16)	Double-blind, randomized, BS: placebo, ketanserin (40 mg, p.o.), Psi (260 mg/kg, p.o.), or Psi + ketanserin	Sensorimotor gating (prepulse inhibition of the acoustic startle response); psychopathological core dimensions (5D-ASC); behavioral inhibition (Stroop task)	Psi: ↓ sensorimotor gating, behavioral inhibition, ↑ all 5D-ASC scores (blocked by ketanserin)	P
Raes et al. (154)	Healthy participants (N(study 1)=164; N(study 2)=39; n(MBCT)=18; n(waiting list)=21)	Study 1: Cross-sectional design to examine the relationship between trait mindfulness and CR; study 2: 8-week MBCT programme or waiting list; BS	Mindfulness (KIMS), CR (LEIDS-R, BDI, MDQ)	Trait mindfulness is negatively correlated with CR; MBCT: ↓ CR (mediated by a positive change in mindfulness skills)	MM
Roseman et al. (155)	Treatment-resistant depression patients (moderate to severe) (N=20)	Open-label, WS, 2 sessions: Psi (10 mg/kg p.o.) and (25 mg/kg p.o.)	Depression (HAM-D, BDI); Amygdala activity to emotional faces (fMRI); Treatment response (in-scanner rating of depressed mood; QIDS)	Psi: ↓ depressive symptoms, ↑ responses to fearful and happy faces in the right amygdala; amygdala(r) increases to fearful versus neutral faces were predictive of clinical improvements at one week	P
Roseman et al. (156)	Healthy participants (N= 40; N(Psi)= 15; N(MDMA)=15)	Pooled data analysis; data from 2 previously published BOLD-weighted fMRI data sets after placebo-controlled administration of Psi (N = 15) and MDMA (N = 25)	Resting-state functional connectivity (RSFC); Subjective effects	Psi> MDMA: changes in consciousness. Psi ↑ between-network RSFC and ↓ RSFC between visual and sensorimotor resting state networks. MDMA had a notably less marked effect on between-network RSFC	P
Ross et al. (97)	Patients with cancer-related anxiety and depression (N=29)	Double-blind, WS, 2 sessions: Psi (0.3 mg/kg, p.o.) and niacin (p.o.), both + psychotherapy	Anxiety and depression (HADS, STAI, BDI)	Psi: anxiolytic and anti-depressant effects which sustained for 6.5 months; effects were mediated by P-induced mystical experience	P
Sampedro et al. (157)	Healthy participants with prior experience with ayahuasca (N=16)	Open-label, uncontrolled, 1 session: ayahuasca (148 mL; 0.3 mg/ml DMT)	Neurometabolic and connectivity modifications (magnetic resonance spectroscopy); mindfulness (FFMQ, EQ, Self-Compassion questionnaire); acute subjective drug effects (HRS)	↓ Neurometabolism in PCC; ↑ PCC-ACC connectivity; ↑ ACC-right MTL connectivity, correlating with ↑ self-compassion; ↓ glutamate + glutamine, correlating with ↑ nonjudging	P
Smigielski et al. (158)	Healthy, experienced meditator subjects (N=38; 23 males)	Five-day mindfulness retreat; Single dose of PSI (0.315 mg/kg, p.o.)	Pre- and post PSI brain dynamics during resting state and two meditation forms	Decoupling of mPFC and posterior cingulate cortices: associated with PSI-induced subjective ego dissolution The extent of ego dissolution and brain connectivity predicted positive changes in psycho-social functioning of participants 4 months later PSI + MM facilitated neurodynamic modulations in self-referential networks	P and MM
Stroud et al. (159)	Patients with treatment-resistant depression (n=17) and controls (n=16; N=23)	BS (patient/control)+WS, 2 sessions: Psi 10 mg/kg, p.o. and 25 mg/kg, p.o.	Depression (QIDS); emotional processing (Dynamic Emotional Expression Recognition Task); anhedonia (SHAPS)	Baseline: patients < controls emotion recognition speed; Psi: ↑ emotion recognition speed compared with baseline in patient (correlated with ↓ anhedonia), but not controls	P
Studerus et al. (160)	Healthy participants (N= 110)	Pooled data analysis; Eight double-blind placebo-controlled experimental studies conducted between 1999-2008; 1–4 administrations of Psi (45–315 mg/kg p.o.)	Psychedelic experience (5D-ASC); Mood (AMRS), long-term drug effects (investigator-constructed follow-up questionnaire)	Psi dose-dependently induced changes in mood, perception, thought and self-experience; most participants described the experience as pleasurable, enriching and non-threatening.	P
Surawy et al. (161)	Chronic fatigue syndrome patients waiting to receive cognitive behavior therapy (N=9)	WS; 8-week MM programme	Fatigue (Chalder fatigue scale); Physical functioning (SF-36); Anxiety/Depression (HADS); Effect of fatigue on QOL (FIS)	↓ fatigue, anxiety, depression; ↑ QOL, physical functioning; effects were sustained for 3 months	MM
Soler et al. (162)	Healthy participants with no prior meditation experience (N=20; n(ayahuasca)=10; n(MBSR)=10)	WS+BS(ayahuasca/MBSR); 4 closely spaced consecutive ayahuasca sessions or 8-week MBSR programme	Mindfulness (FFMW, EQ)	MBSR > ayahuasca: ↑ mindfulness; MBSR = ayahuasca: ↑ acceptance	P and MM
Szabo et al. (163)	Human primary moDCs and autologous naïve T cells	In vitro pre-treatment with DMT and a sigmar-1 agonist, followed by inflammatory response induction with LPS (500 ng/mL), polyI:C (20 µg/mL) or pathogen-derived stimuli versus resting state	Cytokines; gene expression; protein expression	DMT: ↓ inflammatory responses, ↑ anti-inflammatory responses in LPS or polyI:C-stimulated moDCs through the sigma-1 receptor	P
Turton et al. (164)	Healthy psychedelic-experienced volunteers (N= 15)	Qualitative study; Psi (2 mg, IV)	Psychedelic experience (Semi-structured interview)	Reports of perceptual changes (visual, auditory and somatosensory distortions), cognitive changes, mood changes, spiritual or mystical type experiences	P
Vollenweider et al. (165)	Healthy participants (N=10)	WS, 3 sessions: Psi (15, 20 mg) and placebo	CMRglu (PET, FDG); ego pathology (EPI); psychopathology (AMDP); subjective drug effects (APZ)	Psi: global ↑ CMRglu, especially in PFC, ACC, and temporomedial cortex and putamen (correlated positively with hallucinatory ego disintegration)	P
Wachs and Cordova (166)	33 married couples (N= 66)	No intervention	Mindfulness (MAAS); Emotional skills and traits (TAS-20, IRI, SECS, ECQ); Marital quality (DAS, Marital Satisfaction Inventory—Revised)	Mindfulness was associated with marital quality and partners’ emotion skills. The association between mindfulness and marital quality was mediated by emotion repertoire skill.	MM
Winnebeck et al. (167)	Depressed patients with a chronic/recurrent lifetime history (N=74)	BS; brief MBI or control condition	Depression (SCID, BDI); ruminative tendencies (RSQ); mindfulness (FFMQ); Cognitive reactivity to sad mood (LEIDS-R)	MBI: ↑ mindfulness, ↓ ruminative tendencies, cognitive reactivity; MBI > control ↓ depressive symptoms	MM
Zeidan et al. (74)	Healthy participants, no prior meditation experience (N=63)	BS, 4 sessions: MM training (group 1) or listening to a recorded book (groups 2)	Mood (POMS); Mindfulness (FMI); Depression (CES-D); anxiety (STAI); Verbal fluency (Controlled Oral Word Association Test); Visual coding (DSST); WM (DSST, N-back task); Immediate memory span (forward/backward digit span); Information processing speed and attention (N-back task)	Group 1 and 2: ↑ mood; MM training: ↓ fatigue, anxiety, ↑ increased mindfulness, visuo-spatial processing, WM, executive functioning	MM
Zeidan et al. (168)	Healthy undergraduate students with no prior meditation experience (N=82)	BS, 3 sessions: MM training (group 1), sham MM training (group 2), or control condition (group 3)	Mood (POMS); Anxiety (STAI); Heart rate	Group 1 > group 2-3: ↓ negative mood, depression, fatigue, confusion, heart rate	MM

Footnote to clarify the used abbreviations: (R)-DOI, 2,5-Dimethoxy-4-iodamphetamin; 5D-ASC, 5-Dimensional Altered State of Consciousness Rating Scale; 5-HT, Serotonin; ACC, Anterior cingulate cortex; AMDP, Inventory of the Association for Methodology and Documentation in Psychiatry; AMRS, Adjective Mood Rating Scale; ANT, Attention Network Test; APZ, Altered States of Consciousness; ARCI, Addiction Research Center Inventory; ASPIRES, Assessment of Spirituality and Religious Sentiments; BDI, Beck Depression Inventory; BI, Backward inhibition; BPRS, Brief Psychiatric Rating Scale; BSI, Brief Symptoms Inventory; CAR, Cortisol awakening response; CAS, Chinese Affect Scale; CBF, Cerebral blood flow; CEN, Central executive network; CES-D, Center of Epidemiological Studies Depression Scale; CMRglu, Cerebral metabolic rate of glucose; CR, Cognitive reactivity; CPT, continuous performance test; CRP, C-reactive protein; CRS, Competitor Rule Suppression; DAS, Dyadic Adjustment Scale; DASS-21, Depression Anxiety Stress Scale; DERS, Difficulties in Emotion Regulation Scale; DHEAS, Dehydroepiandrosterone-sulfate; DMN, Default-mode network; DMT, N,N-Dimethyltryptamin; DSST, Digit symbol substitution task; DXM, Dextromethorphan; ECQ, Emotional Control Questionnaire; EEG, Electroencephalogram; EORTC QLQ-C30, European Organization for Research and Treatment of Cancer Quality of Life Questionnaire; EPI, Ego Pathology Inventory; EPT, emotion-processing task; EQ, Experience Questionnaire; ESM, Experience Sampling Method; FACIT, Functional Assessment of Chronic Illness Therapy; FAM, Focused Attention Meditation; FAIR, Frankfurt Attention Inventory; FDG, [F-18]-fluorodeoxyglucose; FERT, Facial Emotional Recognition Task; FFMQ, Five Facet Mindfulness Questionnaire; FFNJ, Measure of being nonjudgmental adapted from factor five; FIS, Fatigue Impact Scale; FMI, Freiburg Mindfulness Inventory; fMRI, Functional Magnetic Resonance Imaging; FNE, Fear of Negative Evaluation Scale, GAF, Global Assessment of Functioning; GRE, Graduate Record Examination; HADS, Hospital Anxiety and Depression Scale; HAM-A, Hamilton Anxiety Scale; HAM-D, Hamilton Depression Scale; HRS, Hallucinogen Rating Scale; IFNγ, Interferon gamma; IL, Interleukin; IMS, Investment Model Scale; IRI, Interpersonal Reactivity Index; KIMS, Kentucky Inventory of Mindfulness Skills; LAP, Life Attitude Profile; LEIDS-R, Leiden Index of Depression Sensitivity; LKM, Loving-kindness meditation; LOT-R, Life-, Orientation Test—Revised; LPS, Lipopolysaccharides; LSD, Lysergic acid diethylamide; MAAS, Mindful Attention Awareness Scale; MACAM, Measure of Awareness and Coping in Autobiographical Memory; MADRS, Montgomery–Åsberg Depression Rating Scale; MBI, Mindfulness-Based Intervention; MBI-ld, low-dose MBI; MBSR-C, MBSR programme for cancer; mCPP, m-chlorophenylpiperazine; mDES, Modified Differential Emotions Scale; MDT, Moral Dilemma Task; MET, Multifaceted Empathy Test; moDCs, Monocyte-derived dendritic cells; MOS, Medical Outcomes Studies Short-Form General Health Survey; mPFC, Medial prefrontal cortex; MSAS, Memorial Symptom Assessment Scale; MSCL, Medical Symptom Checklist; MSI-BPD, McLean Screening Instrument for BPD; MTL, Medial temporal lobe; NEO-PI, Big Five Personality Inventory; NK, Natural killer; NMR-15, Negative Mood Regulation Expectancies scale; OSPAN, Operation Span Task; PANAS, Positive and Negative Affect Schedule; PCC, Posterior cingulate cortex; PCPA, P-chlorophenylalanine; PCT, Picture Concept Test; PEQ, Persisting effects questionnaire; PFC, Prefrontal cortex; PHA, Polyhydroxyalkanoate; PILT, Purpose in Life Test; PLMT, pattern/line meanings test; PLOT, Penn Line Orientation Test; POMS, Profile of Mood States; PSQI, Pittsburgh Sleep Quality Index; PSS, Perceived Stress Scale; PWBS, Psychological Well-Being Scale; QIDS, Quick Inventory of Depressive Symptoms; QOL, Quality of Life; RCOPE, Religious Coping Activity Scales; ReSSI, Relapse Signature of Suicidality Interview; RMBPC, Revised Memory and Behavior Problems Checklist; RRQ, Rumination–Reflection Questionnaire; RSQ, Response Styles Questionnaire; SCID, Structured Clinical Interview for DSM-5; SCL-90, Symptom Check List-90; SCS, Self-Control Scale; SDS, Self-Rating Depression Scale; SECS, Self‐Expression and Control Scale; SF-36, Short Form 36; SHAPS, Snaith-Hamilton Pleasure Scale; SOCQ, Selection-Optimization-Compensation Questionnaire; SOSI, Symptoms of Stress Inventory; STAI, State-Trait Anxiety Inventory; SWLS, Satisfaction with Life Scale; TAU, Treatment as usual; TAS-20, Toronto Alexithymia Scale; TMMS, Trait Meta-Mood Scale; TNF-α, Tumour necrosis factor alpha; WM, Working memory.

## Psychological Factors

As put forward in the *Introduction*, the psychological deficiencies of MDD feature a rigid, negatively biased cognitive style that contributes to the recurrence of depressed mood, regulatory difficulties, and social conflict (4, 7, 16). Below, studies describing the effects of MM and psilocybin on mood, executive functioning, and social skills are summarized.

### Mood

MM has been found to elevate mood in healthy participants (74, 108, 168), depressed patients (131, 167) and other conditions (114, 161). For MBSR, this effect was already apparent after training units as short as three days (168) and remained up to six months (114), while MBCT demonstrated sustained effects for three months (161). Studies of mindfulness training that was not combined with psychotherapy only demonstrated short-term mood enhancement in healthy volunteers (74, 108).

It has been proposed that such mood enhancement arises from the acquisition of mental strategies. Mindfulness-based mental strategies are thought to reduce cognitive reactivity, the tendency to engage in negative thinking in response to mildly dysphoric mood, and promotes emotion acceptance, ultimately improving affect regulation (133, 154, 167, 169). Additionally, Huffziger and Kuehner (131) showed that by encouraging non-judgemental awareness of negative thoughts in depressed patients, the association between negative thoughts and negative mood might diminish, and the perpetuation or relapse of depressive symptoms prevented (131). This is in line with the negative association between mindfulness and rumination, and the positive relationship between mindfulness and nonattachment found in healthy volunteers. The latter indicates the degree to which an individual perceives happiness as independent from external circumstances, such as financial wealth or daily-life experiences (117). It is suggested that mindful individuals ruminate less and are consequently less likely to adapt their intrinsic state to affectively salient events in their environment (117, 131).

Psilocybin has been shown to acutely enhance mood in healthy participants (69, 96, 124, 134, 135, 151) as well as depressed (63, 103, 155) and cancer patients (96, 97, 99). This occurred after one to two fixed (10 and 25 mg, p.o.) or weight-adjusted doses (range between 1-30 mg/70 kg, p.o.), and in one study, the improvement was still significant at a 14-month follow-up with 0.2 mg/kg (14 mg/70 kg, p.o.) (99). Several lines of evidence further suggest that the mood-enhancing effect of psilocybin is dose-dependent (93, 88, 89, 128).

The acute effects of psilocybin occasionally involve a “peak” experience, a blissful sense of sacredness, revelation, transcendence of time and space, or connectedness with the environment (170). This often entails psycho-spiritual insights that are reported to be of major personal value and have an enduringly positive impact on well-being, attitude and personality (69, 97, 103, 124, 144). These persisting positive (mood) effects and relative freedom from worry is also called “afterglow” and indicated as an important timeframe for psychotherapeutic interventions (171, 172).

In summary, MM and psilocybin both induce positive mood changes which might outlast the acute MM or psilocybin stage. Effects of both seem to be “dose”-related with more extensive MM practice, and higher doses of psilocybin having more pronounced effects. Nonetheless, we suggest that MM and psilocybin have a different mechanism of action to induce the same effect. For MM, repeated training promotes the use of mental strategies, altering the cognitive frame in which negative thoughts are perceived and coped with (e.g., 133). With regard to psilocybin, perceptual and thought contents are directly altered by destabilizing established belief systems resulting in a restoration of adequate mood regulation (e.g., 69, 172, 173).

The combination of the strategy-based approach of MM with psilocybin’s content-based approach (**Figure 2**) could possibly contribute to a potentiation or longer maintenance of induced mood enhancements. A reduction of cognitive reactivity and promotion of emotional acceptance through MM practice may prevent a relapse of negative thought patterns when the afterglow subsides. Also, Griffiths et al. (69) found that more extensive spiritual practice, including meditation, was associated with a higher frequency of psychedelic-induced peak experiences. This implies that MM practice might be able to facilitate peak experiences, which would result in an increased likelihood of personal insights and enduring positive mood effects. Likewise, positive mood state following personal insights during a psychedelic experience might, facilitate the non-judgmental observation of negative thoughts, since previous research suggests a bidirectional positive relationship between positive mood and mindfulness (121).

Whether the single or combined practice in psychiatric patients would be beneficial is another question. Two recent studies investigating the effects of ayahuasca, another psychedelic substance with similar 5-HT2A agonistic action as psilocybin was shown to increase emotion regulation and some aspects of mindfulness in healthy volunteers (162, 118). Of note, mindfulness was not increased in participants with borderline personality disorder traits (118). This may bear meaningful clinical implications, as people with certain psychopathologies, including depression, might be less receptive to a psychedelic-induced enhancement of mindfulness.

### Executive Functioning

Studies have demonstrated positive effects of MM on executive functioning, including improvements in cognitive flexibility (122). Repeated training of mindfulness has been shown to improve WM as well as attentional and inhibitory capacities (74, 123, 132, 147, 148).

A possible explanation for MM-induced executive function enhancement builds on an incremental reduction of mind-wandering together with an enhancement of meta-awareness. The former describing the tendency to drift off with one’s thoughts, while the latter can be conceptualized as the acknowledgement of ongoing mental processes, which shares a neural signature with that of executive functions. Accordingly, this hypothesis was supported empirically (74, 126, 149). A positive “side” effect of MM’s cognition-enhancing effects is a more pronounced subjective sense of control, as shown in a study investigating the effects of MBSR (105).

In contrast to MM’s homogenously positive effects across cognitive domains, psilocybin tends to acutely impair some aspects of executive functioning like inhibition, attention, and WM (93, 115, 116, 153), while improving others by, for example, inducing a greater bias towards positive stimuli (134), or leaving some processes unaffected, like spatial WM (107, 115, 116, 153).

The feeling of loss of control over thoughts or perceptions is frequently reported in psilocybin trials, which is linked to adverse reactions, and may reflect the induced decreases in executive control (128, 160, 164). On the other hand, psilocybin’s dys-executive effects have also been proposed to offer therapeutic implications as to surface suppressed emotions and thoughts in order to confront them and, hence, restore emotional responsiveness in MDD (155).

Further, consistent with the notion that psilocybin impairs cognitive focus and control, 5-HT2A agonism has been implicated in an acute decline in convergent thinking, which critically relies on adequate executive functioning (137). In line with this, it was shown that LSD-induced impairment of working memory, executive functions, and cognitive flexibility was mediated by the 5-HT2A receptor (174). Conversely, 5-HT2A agonism is also associated with increased cognitive flexibility and divergent thinking (106, 119, 127, 137, 144, 175). The latter findings might be suggested to underlie decreased executive control and a loosening of associations *via* neuroplastic changes in core neural networks, although this hypothesis has to be tested. Additionally, it is suggested that these psychedelic-induced increases in cognitive flexibility are potentially long-lasting (119, 127, 144).

Taken together, MM demonstrates relatively global cognition-enhancing effects upon repeated training, whereas psilocybin’s effects on executive functioning build on increased acute disinhibition and enduring cognitive flexibility (**Figure 2**). Supporting psilocybin-assisted therapy with MM practice may have the potential to buffer feelings of loss of control associated with the acute psychedelic effects by boosting both subjective and objective executive functioning and, consequently, reducing the risk of adverse reactions. Alternatively, an interference with the individual effects of either treatment is also possible. For instance, by improving cognitive control, MM may reduce psilocybin’s cathartic effects or, conversely, psilocybin might exacerbate the practice of MM during acute effects, as certain aspects of mindfulness, especially in FAM, build on attentional capacities (139).

### Social Skills

With regard to social skills, MM has been linked to greater relationship satisfaction, as it supposedly fosters a more adequate expression and recognition of feelings and reduces the degree to which an individual is emotionally affected by distressing social events (107, 166, 169). This led to the emergence of variants of MBIs that specifically focus on the interpersonal aspects of MM, such as mindful relating (166) or mindfulness-based relationship enhancement (MBRE) (176). These trainings are encompassed by the frame term “relational mindfulness” and lay emphasis on fostering compassion and attentive communication to others.

Psilocybin has positive acute and subacute effects on some aspects of empathy (95, 151, 177). A recent study (159) further showed that psilocybin improved emotional face recognition (cognitive empathy) in TRD patients, while another study demonstrated reduced feelings of social exclusion and in healthy volunteers following psilocybin administration compared to placebo (153).

Pahnke (171) suggested that, in the afterglow, the willingness “to enter into close interpersonal relationships” may be heightened, which is in agreement with self- and other reports of positive changes in social attitudes and behavior following psychedelic peak experiences (69, 88, 96, 102). One aspect of peak experiences in particular, namely the phenomenon of ego dissolution, could be meaningful in this context. Ego dissolution can be described as the loss of sense of identity that is separate from its surroundings and is, therefore, accompanied by an intense feeling of connectedness with the environment. Such an experience may contribute to the destabilization of self-centered belief systems and open the individual up to his or her social surroundings (172). This theoretical implication is supported by the finding of enduring increases in the personality trait “openness” following a psilocybin session (69, 144).

Both MM and psilocybin appear to induce long-term enhancements of social skills. Based on studies conducted up until now it is suggested that MM does so by influencing the way an individual deals emotionally with social encounters, which, as a result, promotes adequate social behavior, or interpersonal social skills. Psilocybin seems to predominantly act on an intrapersonal level of social skills by means of enhanced empathic abilities and a changed personality (**Figure 2**). Combining MM and psilocybin could potentially enhance social relationships more efficiently, as changes in social cognition effectuated by psilocybin would be expected to complement changes in social behavior induced by MM.

## Biological Factors

Studies have shown a range of biological deficiencies to be implicated in MDD among which impaired neuroplasticity, an imbalance in core neural networks, and disturbances in stress responses which are visible as disruptions in neuroendocrine and neuroimmune parameters (21, 22, 29). In the next section MM and psilocybin effects on these processes are summarized.

### Neuroplasticity

For MM, BDNF-promoting effects appear to be linked to prolonged, repeated practice (110) rather than a single, brief training session (125). The exact mechanisms underlying these effects are unclear, though. While MM’s relation to serotonin signaling has not been investigated yet (101), the expression of BDNF may be enhanced by either frontal activation following active engagement of attention, vagal stimulation or a reduction in stress response (110).

Psilocybin and related classical psychedelics, such as lysergic acid diethylamide (LSD) or N,N-dimethyltryptamine (DMT), are hypothesized to promote neuroplasticity through mechanisms involving 5-HT2A agonism (67). Serotonin 2A receptors, to which psilocin binds, are especially prominent on large glutamatergic pyramidal neurons in deep cortical layers projecting to layer V pyramidal neurons of the PFC, and on layer V itself. These receptors are suggested to rapidly increase in activity as psilocybin is ingested, hypothetically resulting in an elevated expression of BDNF (67, 143). The resulting temporarily state of heightened neuroplasticity may already occur after a single, psychotropic dose and could allow for major synaptic changes, which was suggested to offer an important opportunity for psychotherapeutic interventions (67).

This indicates that the effects of psilocybin and MM on neuroplasticity differ in aetiology and magnitude. Psilocybin could induce a transient, but powerful neuroplastic boost, which is driven by bottom-up glutaminergic processes. In contrast, MM supposedly relies on top-down regulatory efforts and encourages plasticity incrementally throughout the progress of training (**Figure 2**). These approaches may support one another, as MM could possibly serve to prolong the potential neuroplastic state induced by psilocybin and psilocybin might boost the rate at which BDNF rises throughout MM training.

### Neural Core Networks

MM has differential effects on the SN and CEN with SN regions, the insula and anterior cingulate cortex (ACC), being engaged during mediation, whereas the activity of CEN regions, the lateral PFC and parietal cortex, decreases (75). As the insula and ACC are involved in interoceptive processes (178) and the lateral PFC and parietal cortex in external awareness (179), this is thought to reflect inward-focused attention during the practice of mindfulness (75, 77). Moreover, MM promotes the activation of the dorsolateral PFC, a key region of the CEN, which is important for cognitive control (142, 180). Long-term meditators show increased cortical thickness in the insula, sensory cortices, and PFC as well as reduced volume of the amygdala, a region involved in fear responses (129, 138). This supposedly represents decreased emotional over-reactivity and increased regulatory control that has been manifested through repeated practice, which is in line with the effects on mood and executive functioning, as discussed above.

Psilocybin was proposed to globally decrease functional neural integrity within, while increasing connectivity between networks, which may be responsible for the experience of hallucinations, loosening of strong associations, and increases in cognitive flexibility following its administration (112, 156, 175). Most notably, the cortical disintegration of the DMN has been implicated in the occurrence of social skill-related ego dissolution and increases in some aspects of mindfulness (77, 157, 181). In addition, an increased functional connectivity between SN and CEN contrasts the aforementioned effects of MM on these networks (156), which might relate to the treatments’ opposing cognitive effects. The glutaminergic action of 5-HT2A receptors discussed in the previous section would suggest that psilocybin induces widespread cortical activations, particularly in association cortices, where 5-HT2A receptors are most abundant (182). Consistent with this expectation, some studies appear to endorse acute psilocybin-induced hyper-activation in frontal regions, as opposed to more posterior regions, and this pattern of activity correlated positively with the measures of psychotic symptoms, especially ego dissolution (106, 141, 165). An fMRI study of psilocybin’s acute effects showed deactivations in cortical hub regions, such as the posterior cingulate cortex and thalamus. This could be explained by an involvement of GABAergic interneurons within psilocybin’s pathway of action, which, when excited, inhibits subsequent neurons (111). The apparent paradox between the frontal hyper-frontality shown in one study (165) and the decreased perfusion in frontal regions by another study (111) was suggested not to be in contrast, but rather dependent on the method of analysis (141). It was suggested to interpret the relative changes in perfusion in relation to absolute signal variations, and to report two analyses, with and without this “correction” for global activity as a solution to enhance transparency, reduce inconsistencies, and help in the interpretation of findings (141). Nonetheless, as cortical hubs play a crucial role in coordinating the flow of information across functionally discerned brain areas, their inhibition might result in sub-optimal communication between brain areas involved in executive control, reflecting the disinhibition effects of psilocybin (183).

While jointly working to resolve DMN dominance associated with excessive rumination (22, 23, 77), MM and psilocybin seem to alter circuits differentially. MM additionally targets areas related to interoception and executive control, while psilocybin has a more wide-spread effect on functional integrity, potentially promoting flexible cognition (**Figure 2**). This appears to reflect the MM- and psilocybin-induced psychological changes described earlier. By reorganizing the connectivity between the DMN, CEN and SN, MM, and psilocybin may restore normal functional integration in patients, which could contribute to a reduction of negative and rigid thinking patterns. Relevant in this light is the recent study by Smigielski et al. (158) who administered a single dose of psilocybin (0.315 mg/kg, p.o.) to healthy, experienced meditators, during a five-day mindfulness retreat. The pre-post brain resting state analysis revealed a decoupling of medial prefrontal and posterior cingulate cortices, which was associated with the psilocybin-induced subjective ego dissolution. Of note, the extent of ego dissolution and brain connectivity predicted positive changes in psycho-social functioning of participants 4 months later.

### Neuroendocrine and Neuroimmunological Factors

The attenuation of stress responses has been suggested to be a central mechanism through which MM exerts its beneficial effects on mental and physical health. MM may do so by, reducing stress-reactivity, in addition to promoting regulatory prefrontal pathways, involving a reduction in amygdalar projections and HPA axis activity (68). However, although MM training generally reduces subjective psychological stress (140, 150), its effect on cortisol secretion varies across populations. In healthy volunteers, eight weeks of MBSR training had no effect on cortisol levels (145, 150), whereas, in cancer patients, cortisol levels decreased significantly under comparable intervention settings (113, 140).

As diseases represent sources of profound stress, this may imply that the association between MM and cortisol only holds for highly stressful situations, which was supported empirically by Brown et al. (109). Accordingly, it is conceivable that MM also reduces cortisol levels in depressed patients. Instead, what has been observed by Matousek et al. (146) was that MBSR increased the CAR in cancer patients who demonstrated depressive symptoms. This conforms an alternative hypothesis, namely that MM not merely reduces cortisol, but rather optimizes HPA responsivity (78, 184). However, another study investigating the effect of MBCT on the CAR in patients remitted from recurrent depression did not support the findings by Matousek et al. (146) (120), which may be due to the use of MBCT rather than MBSR (78). MBSR, as opposed to MBCT, is implicated in being a particularly suitable means for diminishing overall stress symptomatology, as it promotes specific stress coping strategies (79, 113). Although MM might additionally reduce pro-inflammatory cytokines, including IL-6, these findings are inconsistent (140, 145), but may originate from vagal stimulation, which is thought to induce a cholinergic anti-inflammatory reflex (185).

Psilocybin, on the other hand, is associated with an acute increase in cortisol levels (128). In accordance with the involvement of cortisol in attention and memory, this transient elevation could possibly facilitate extinction learning of negative associations by prioritizing the formation of new memories over the retrieval of older memories (186, 187).

Moreover, psilocybin reduced subjective stress in terminally ill cancer patients during the first three months following administration (99). An incremental down-regulation of 5-HT2A receptors is suggested to play a role in this as prefrontal 5-HT2A receptors were found to be involved in stress response pathways (188). Furthermore, by activating prefrontal areas, psilocybin might encourage top-down control of stress responses in limbic structures, such as the amygdala (67).

5-HT2A agonism has also been linked to major anti-inflammatory action, as 5-HT2A receptors are integrated in an abundance of cells throughout the immune system (149, 189, 190). Psilocybin and related psychedelics are hypothesized to distort cell signaling within the immune system by selectively stimulating anti-inflammatory pathways (191, 192). Although this is yet to be tested with psilocybin, LSD, DMT, and 2,5-Dimethoxy-4-iodoamphetamine (DOI) were found to have anti-inflammatory action, inhibiting the production of IL-6 (130, 149, 163) which might account for enduring antidepressant psychedelics effects (193). Nonetheless, there is evidence that 5-HT2A receptors are also involved in pro-inflammatory responses. The extent to which 5-HT is immunosuppressive or immune-activating may depend on its blood concentration (136).

The neuroendocrine and neuroimmune system are interdependent networks that communicate by means of hormone and cytokine signaling (194). MM and psilocybin act differentially and possibly complementarily on these systems. Through the progressive strengthening of regulatory control and reduction of stress-responsiveness, MM optimizes HPA axis functioning and may, eliminate immune system disruptions. Psilocybin, one the other hand, could transiently reduce inflammatory responses by means of 5-HT2A agonism and consequently reduce the stimulation of the HPA axis through anti-inflammatory cytokines (**Figure 2**).

## Discussion

Depression is a major public health problem, to which conventional treatments represent an insufficient solution (1, 45, 51). MM and psilocybin appear to be promising novel treatments, and combined their resulting therapeutic effect might even be greater. However, the current literature is limited to theoretical and empirical underpinnings of their singular use in treatment (e.g., 61, 63). The present review therefore aimed to identify possible additive or complementary effects of MM and psilocybin on six factors (mood, executive functioning, social skills, neuroplasticity, neural core networks, neuroendocrine, and neuroimmunological factors) associated with MDD in order to offer theoretical implications for future clinical research of depression. Findings showed that MM and psilocybin exerted similar effects on mood, social skills, and neuroplasticity; different effects were found on executive functioning, neural core networks, and neuroendocrine and neuroimmune system markers. The effects on mood were “dose”-dependent, with more MM practice or higher psilocybin doses leading to more pronounced mood effects; effects on neuroplasticity were already visible after a single dose of psilocybin, while more MM practice sessions were needed before effects were visible. While for most factors the combination of MM and psilocybin is potentially beneficial, this was not clear for executive functions.

From a psychological perspective, MM employs mental strategies that augment emotional and cognitive self-regulation in the long term (73, 133, 154), whereas psilocybin has neuromodulatory effects that induce a state of apparent “flexible” cognition, and may lead to personal insights that diminish negative biases (102, 111). A combination of MM and psilocybin could possibly shift both the cognitive frame and content of thoughts towards a more positive, open-minded outlook, promote the feeling of control over strong emotions that might occur under the acute effects of psilocybin, or improve communication skills. This may ultimately enhance psychological factors, such as mood, cognitive control, and relationship satisfaction. Recent research suggests that the extent of psilocybin-induced ego dissolution during a mindfulness session might play a very important role in the endurance of positive changes in psycho-social functioning (158).

From a biological perspective, MM serves to adjust prefrontal and limbic activity and HPA reactivity through repeated top-down control (129, 138, 184). Psilocybin, on the other hand, promotes global network disintegration and anti-inflammatory effects involving transient bottom-up processes (175, 191). Pairing these effects may result in a two-way reorganization of neural networks, especially those involved in rumination, and downregulation of neuroendocrine and neuroinflammatory responses. Part of this suggestion was investigated and supported by a recent study that showed decoupling in self-referential networks and the psilocybin-induced change in self-experience, during a mediation retreat, to be predictive of enduring positive changes in psycho-social functioning (158). Together these findings offer several implications for future clinical research into MDD.

### Implications for Future Research

The present findings suggest that the combination of MM and psilocybin could possibly exert larger or longer-lasting effects in the treatment of MDD than either treatment alone. These effects may particularly relate to enhancements in mood, social skills, neuroplasticity, and a reduction of stress-related neuroendocrine and neuroinflammatory markers. Testing this hypothesis requires comparisons of changes in these variables in depressed patient groups in a—preferably—randomized, double-blind, placebo-controlled trial with repeated measurements to test acute and persistent effects, weeks to months after treatment.

Ideally, psilocybin-assisted MBI is compared to psilocybin and MM alone, and to a conventional antidepressant (SSRI). To test the effects of MBI on the variables of interest a “psychological” placebo, e.g., minimal psychological support based on CBT principles, complementing the pharmacological manipulation, is needed. This is also warranted since the administration of psilocybin without psychological support is not recommended (94). Primary endpoints would focus on depressive symptomatology assessed with daily diaries and weekly assessments with the Hamilton Depression Inventory or the Beck Depression Inventory (BDI) (195–197). Secondary endpoints would be social skills and executive functioning, assessed with cognitive tests, self-reports, and structural and functional brain imaging; neuroplasticity (BDNF), neuroendocrine (cortisol, oxytocin) and neuroinflammatory (cytokines) factors assessed in blood samples. To add, self-reports from patients and observational reports from significant others could be used to test whether depressed patients indicate less conflict and higher relationship satisfaction following a psilocybin-assisted MBI than their respective control groups as both treatments are known to alter the perception of social relations (107, 172). Cognitive tests at different time points in the treatment will be useful to dissociate short- and long-term effects of the combination of MM and psilocybin on executive functioning and clarify potential opposing effects on such processes as suggested by the inconclusive findings in the present review.

To date, no norms regarding the exact procedure, type of psychological support, dose(s) of the psychedelic, or duration of the psychedelic therapy have been agreed upon (198). With regard to the psychological component of psilocybin-assisted MBI therapy, the typical treatment duration of eight weeks MBI may be appropriate (78). To add, it has not yet been determined whether MM should precede or follow the administration of psilocybin. The present findings would endorse MM practice prior to a psilocybin session, as it may have the potential to reduce the risk of adverse effects in depressed patients due to its positive effects on mood and cognition (e.g., 74). Moreover, the findings imply that MM could facilitate the occurrence of peak experiences upon psilocybin administration (69), something that has been shown to be important in the treatment response (97, 155).

Considering the potential benefits of these implications, future studies could test if (eight weeks of) MM practice prior to a psilocybin session can decrease potential adverse reactions such as anxiety, and increase the chance of having a peak experience, or increase the intensity of the experience during a psilocybin session, compared to appropriate control conditions (69, 102, 124, 144).

Lastly, a combination of MM and psilocybin may also bear benefits for MDD patients in a more indirect way, as findings indicate. Mindfulness could represent a useful asset to the training of psychedelic therapists (199, 200). Future studies may test if patients of psychedelic therapists trained in mindfulness demonstrate better outcomes on psychological measures of depression in comparison to patients of therapists that were not trained in mindfulness.

### Limitations

Upon discussing scientific implications that the findings offer, it is important to mention that the present review features a number of limitations. First, due to different methodologies, findings of the included studies are difficult to compare. For example, studies examining the effects of MM have used diverse assessment methods to measure similar variables in different populations, which could explain the inconsistent findings across studies. For instance, while Carlson et al. (114) demonstrated positive effects of MBSR on mood states (Profile of Mood States) of cancer patients compared to pre-MM scores, Astin (105) did not demonstrate significant effects of MBSR on mood (Symptom Check List-90-R (SCL-90-R)) in undergraduate students. While the (physical and mental) difference in groups are apparent, the construct differences between questionnaires might not be that obvious. Whereas the POMS is specifically designed to assess mood states, the SCL-90-R screens for a broad range of clinical symptoms (201–203).

Another example is the significant decrease shown in immune markers (salivary IL-6 level) in healthy participants (cancer patient caregivers) after six weeks of MBSR, and the absence of this finding in university staff and students following an 8-week-long low-dose MBI (145). Despite both groups being regarded as healthy, it is apparent that they were exposed to dissimilar kinds of stressors, which precludes inferences about general effects of MM on immune system markers.

Another methodological issue noted in the reviewed MM studies is the general lack of active control groups, which impedes the differentiation of effects that are specific to MM from those that apply to any other psychological treatment. Hence, points of attention when conducting a study investigating the effects of MM are to use gold standard tests to assess certain constructs and the inclusion of active control groups (203).

As for studies investigating the effects of psilocybin there are a number of methodological issues that at this moment withhold from making firm statements about potential implications. Examples are the small number of patients samples (96, 99, 103, 159), the use of an open-label design, and no control group (103). These methodological choices make the generalization of findings to larger populations not possible at this stage, and due to the use of open-label or uncontrolled designs, pharmacological effects cannot be separated from expectancy or placebo effects. Additionally, psilocybin is routinely combined with psychological support, making it difficult to dissociate the psychotropic from general care effects (62, 63, 69).

Moreover there are conceptual issues regarding the definition of MM, as it comprises various forms, such as FAM, MBCT or MBRE. These techniques emphasize different aspects of mindfulness and consequently yield diverse psychological and biological effects (60, 75, 176). For example, the effect of MM on cortisol (CAR) differs between MBCT and MBSR (78, 120). Hence, findings in one study may not necessarily apply to all forms of MM and introduce methodological noise.

With regard to psilocybin, and its mechanism of action, the discussion largely pertained to 5-HT2A agonism (67, 136, 143, 149, 175, 188–191, 204) while psilocybin is also known to act on other neurotransmitter systems (87) which might be relevant for the comparison with MM.

Further, in some of the included papers, hypotheses were proposed that have not been subjected to sufficient empirical testing, such as proposed mechanisms regarding the immunosuppressive action of psilocybin and BDNF-promoting effects of MM (67, 68, 110, 191). To draw definite conclusions, premises based on concrete empirical evidence are needed, and therefore, the inferential power of the present review with regard to the aforementioned is limited. These hypotheses were nevertheless incorporated with other reviewed literature in order to speculate on potential interaction points between psilocybin and MM that may be of value in the treatment of depression upon investigation.

## Conclusion

The present review provides an extensive overview of the current scientific knowledge on the effects of MM and psilocybin on specific pathological depressive features, and on how both interventions might be complementary or even synergistic when combined, in the treatment of depression. With this a valuable theoretical ground for future research is presented. Future studies investigating these effects in both healthy and depressed populations, using rigorous control conditions and representative samples, will provide more knowledge on possible implementation of psilocybin-assisted MBI in clinical practice.

## Author Contributions

KH and KK conceptualized the review question. KH conducted the literature search. KH conceptualized the first version and figures. KH and KK wrote the paper.

## Conflict of Interest

The authors declare that the research was conducted in the absence of any commercial or financial relationships that could be construed as a potential conflict of interest.
